# Effect of Adding Pulses to Replace Protein Foods and Refined Grains in Healthy Dietary Patterns

**DOI:** 10.3390/nu15204355

**Published:** 2023-10-13

**Authors:** Sanjiv Agarwal, Victor L. Fulgoni

**Affiliations:** 1 NutriScience LLC, East Norriton, PA 19403, USA; 2 Nutrition Impact, LLC, Battle Creek, MI 49014, USA; vic3rd@aol.com

**Keywords:** Healthy U.S.-Style Pattern, pulses, lentils, legumes, beans and peas, dietary modeling

## Abstract

Pulses are dry seeds of legumes which are high in fiber and contain plant protein and several important macronutrients. Our aim was to model the nutritional effects of substituting servings of protein foods and/or refined grains with servings of beans and peas in the Healthy U.S.-Style Pattern identified in the *Dietary Guidelines for Americans, 2020–2025*. Dietary modeling was accomplished by substituting nutrients of protein foods and/or refined grains with nutrients of the USDA’s beans and peas (pulses) composite in the 2000 kcal Healthy U.S.-Style Pattern. A 10% or more change was used as an indicator of meaningful differences. Cost implications were computed by adding the cost of pulses and subtracting the cost of protein foods/refined grains according to the modeling scenario. The substitution of 6–8 oz/week protein foods with 1.5–2.0 cups/week pulses increased fiber and decreased cholesterol. Higher amounts of pulses replacing refined grains or combinations of protein foods and refined grains generally increase fiber, iron, magnesium, potassium, and copper depending on the modeling scenarios. All modeling scenarios of substituting the servings of protein foods alone or in combination with refined grains with the servings of pulses were associated with cost savings. Our results suggest that encouraging increased pulse consumption may be an effective strategy for improving diet.

## 1. Introduction

Pulses are the dry seeds of legumes (e.g., dry peas, lentils, chickpeas, and dry beans) which are high in fiber, plant protein, and several important macronutrients, which are important components of healthy diets. Pulses are defined as dry harvested legumes, beans, peas, lentils, and chickpeas; however, legumes with a high amount of moisture or fat/oil at harvest, such as fresh beans, peas, edamame, and peanuts, are not considered as part of pulses [1,2,3,4]. Pulses are a nutrient-dense food that contain many nutrients, such as complex carbohydrate, protein, fiber, folate, iron, magnesium, potassium, choline, zinc, selenium, and phosphorus, and also contain phytochemicals or bioactive nutrients [3,4,5,6]. In a recently published analysis of the National Health and Nutrition Examination Survey (NHANES) data, a regular consumption of pulses was found to improve the diet quality and nutrient density of the diet of U.S. adults [7]. Recent scientific evidence also suggests a beneficial role of pulses in reducing the risk of chronic diseases such as CVD [8,9,10,11], cancer [12,13,14], diabetes [15], and obesity [16,17], and the overall mortality risk [18]. Pulses have also been identified to play a potential role in sustainable food systems [19].

Pulses are consistently included in food-based dietary guidelines globally as part of the vegetable and/or protein food groups or as a separate food group [2,3,20]. In the U.S., the Dietary Guidelines for Americans 2020–2025 (DGA) indicated that the beans, peas and lentils (pulses) can be considered as part of the vegetable or protein food groups, but should be counted in only one food group [21]. The USDA’s Choose MyPlate [22] considers beans, peas, and lentils (pulses) as a part of the vegetable group and also a part of protein foods as they are an excellent source of fiber, folate, and potassium, like vegetables, and are excellent sources of plant protein that also provides iron and zinc like other protein foods. DGA recommend consuming 1.5 cups of pulses per week as part of healthy diets [21]. Specifically, the USDA’s Healthy Dietary Patterns (Healthy U.S.-Style Pattern, Healthy Vegetarian Style Pattern, and Healthy Mediterranean Style Pattern), that were released as part to DGA to help individuals follow the DGA recommendations, include 1.5 cups of pulses per week as part of the vegetables group. In the Healthy Vegetarian Style Pattern, 6 oz eq/week (or 1.5 cups/week) of pulses are also recommended as a part of the protein foods [21,23]. The recent Eat Lancet Commission Planetary Health Diet recommended consuming 100 g/day or 0.5 cups/day (50 g of dried beans, lentils, and peas; 25 g of soybeans; and 25 g of peanuts) as a part of nutritious and environmentally sustainable dietary patterns [24]. However, only ~27% of adult Americans consume pulses on any given day with an average daily consumption of less than 0.5 cup equivalent per day, according to a recent analysis of the NHANES 2013–2014 data [7].

We hypothesize that since pulses have high amounts of protein and are rich in carbohydrate, they could be used as a replacement of animal protein and refined grains. A key objective of current research was to model the effect of increasing the amounts of pulses (dry peas, lentils, chickpeas, dry beans, and canned beans) in the Healthy U.S.-Style Pattern identified in the Dietary Guidelines for Americans 2020–2025 from 1.5 cups/week to up to 3.5 cups/week as a replacement of current protein foods. Additional objectives included examining the impact of pulses replacing refined grains since pulses also contain high amounts of carbohydrates and also simultaneously replace a combination of refined grains and current protein foods.

## 2. Materials and Methods

To achieve the objectives of this study, we used the USDA’s dietary modeling approach [23]. Briefly, the USDA’s Food Patterns provide amounts of foods from the five major food groups and subgroups, including (1) Fruits; (2) Vegetables (dark green, red, and orange, beans and peas, starchy, and other); (3) Dairy (milk, cheese, yogurt, includes calcium-fortified soy beverages); (4) Grains (whole grains and refined grains); and (5) Protein Foods (meats, poultry, and eggs; seafood; nuts, seeds, and soy products). The USDA creates food item clusters of the food groups and then determines the amounts of energy and nutrients that would be obtained by consuming various foods within each group. With this basic information, the USDA then develops dietary patterns with the suggested levels of consumption of each food group/sub-group and confirms that the patterns meet the caloric and nutrient needs for numerous sub-population groups. We chose to focus on making changes to the protein foods consumed (decrease other protein food amounts and increase pulses amount) and/or refined grains (decrease refined grain amounts and replace with pulses). Thus, the following dietary modeling scenarios were used for modeling the 2000 kcal Healthy U.S.-Style Pattern:

1. Replacing 1, 2, 4, 6, and 8 oz eq/week of current protein foods (mix of protein foods: meat, chicken, fish, eggs, nuts, etc.) as provided by the USDA’s Food Pattern Modeling Report [23] with 0.25, 0.5, 1.0, 1.5, and 2.0 cups/week, respectively, of pulses. Since 2000 kcal Healthy U.S.-Style Pattern includes 1.5 cups/week of beans and peas (pulses), this additional 0.25 to 2.0 cups/week would increase the total amount of beans and peas to 1.75 to 3.5 cups/week.
2. Replacing 1, 2, 3, and 4 oz eq of current refined grains as provided by the USDA’s Food Pattern Modeling Report [23] with 0.5, 1.0, 1.5, and 2.0 cups/day of pulses.
3. Replacing the combinations of 2 oz eq/day of current protein foods and 1, 2, and 3 oz eq/day of refined grains with 1, 1.5, and 2.0 cups/day of pulses.
4. Replacing the combinations of 4 oz eq/day of current protein foods and 1, 2, and 3 oz eq/day of refined grains with 1.5, 2.0, and 2.5 cups/day of pulses.

The base nutritional profile of 2000 kcal Heathy U.S.-Style Pattern was obtained from the Food Pattern Modeling Report [23] and is presented in Table 1.

The nutrient profile of the USDA’s Beans and Peas (pulses) composite was obtained from the Food Pattern Modeling Report [23]. The USDA’s Beans and Peas composite (with representative food in parentheses) includes 14.3% black beans (Black, brown, or Bayo beans; dry, cooked, fat not added in cooking); 5.4% chickpeas (Chickpeas; dry, cooked, fat not added in cooking); 0.3% cowpeas (Cowpeas; dry, cooked, fat not added in cooking); 12.1% Kidney beans (cooking); 7.6% lentils (Lentils; dry, cooked, fat not added in cooking); 0.5% lima beans (includes fava and mung beans, lima beans; dry, cooked, fat not added in cooking); 41.9% pinto beans (includes pink beans, yellow beans, Pinto, calico, or red Mexican beans; dry, cooked, fat not added in cooking); 1.3% soybeans/Edamame (Soybeans; cooked, fat not added in cooking); 0.8% split peas (Green or yellow split peas; dry, cooked, fat not added in cooking); 2.7% unknown legumes (White beans; dry, cooked, fat not added in cooking); and 13.0% white beans (White beans; dry, cooked, fat not added in cooking). However, Soybean/Edamame included in the USDA’s composite was in the pods of green legumes (not dried like other beans and peas); therefore, we used a modified USDA’s beans and peas composite which was developed by proportionally subtracting the nutritional contribution of edamame (nutritional profile obtained from the USDA’s FoodData Central, FCD ID: 2342911) [25] and adjusting the calories from the rest of the composite. The nutritional composition of the revised composite without edamame are also presented in Table 1. The amounts of pulses in cups/day used in different modeling scenarios are presented in Supplemental Table S1. 

The protein foods composite in the USDA’s Food Pattern Modeling Report [23] includes 38.21% Red meats, 29.39% Poultry, 2.71% High omega 3 fish, 5.95% Low omega 3 fish, 9.44% Eggs, 1.78% Soy Products, and 12.51% Nuts/Seeds. The nutritional profiles of each component were obtained from the Food Pattern Modeling Report [23] and the nutritional composite of protein foods was computed by adding the nutrients of each individual component in the above proportion and is provided in Table 1. The nutrient profile of Refined Grains was also obtained from the USDA’s Food Pattern Modeling Report [23] and is also provided in Table 1.

Dietary modeling was accomplished by substituting the nutrients from protein foods and refined grains (alone or in combination) with the nutrients from pulses in the desired amounts and modified nutrient profiles were created using Microsoft Excel (Version 2019, Microsoft, Inc., Redmond, WA, USA). A change of 10% or more in nutrients due to the dietary modeling analyses of the 2000 kcal Healthy U.S.-Style Pattern was used as an indicator of meaningful differences.

The cost of beans and peas (pulses) replacing current protein foods and/or refined grains was estimated by linking the Purchase to Plate National Average Prices (PP-NAP) database [26] (from the USDA’s Economic Research Service) for 2015–2016 with the food codes from the Food Patterns Equivalent Database (FPED) 2015–2016 [27] for the representative foods provided in the Food Modeling Report [23]. Cost implications were computed by adding the cost of pulses and subtracting the cost of protein foods/refined grains according to the modeling scenario.

## 3. Results

Substituting 6 and 8 oz eq/week of protein foods with 1.5 and 2 cups/week of beans and peas (pulses) without edamame increased fiber and decreased cholesterol by more than 10% from the baseline. However, changes in the other nutrients did not reach our a priori defined 10% cutoff point. Substituting protein foods with lower amounts of pulses did not result in changes in any nutrients by more than 10% from the baseline (Table 2).

Substituting 1 oz eq/day of refined grains with 0.5 cups/day of pulses increased fiber, magnesium, potassium, and copper by more than 10% from the baseline; substituting 2 to 4 oz/day of refined grains with 1 to 2 cups/day of pulses increased protein, fiber, iron, magnesium, phosphorus, potassium, zinc, copper, vitamin B_6_, and choline, and decreased sodium by more than 10% from the baseline (Table 3). Additionally, replacing 3 and 4 oz eq/day of refined grains with 1.5 and 2.0 cups/day of pulses also increased vitamin E and decreased niacin by more than 10% from the baseline. Selenium also decreased by more than 10% from the baseline in replacing 4 oz eq/day of refined grains with 2 cups/day of beans and peas without edamame (Table 3).

Substituting 2 or 4 oz eq/day of protein foods in combination with 1 to 3 oz eq/day of refined grains with 1.0 to 2.5 cups/day of pulses consistently increased carbohydrate, fiber, iron, magnesium, potassium, and copper, and it decreased cholesterol, sodium, selenium, niacin, and vitamin B_12_ by more than 10% from the baseline (Table 4). Substituting 4 oz/day of protein foods in combination with 1 to 3 oz eq/day of refined grains with 1.5 to 2.5 cups/day of pulses additionally increased thiamine and decreased total fat, saturated fat, and riboflavin by more than 10% from the baseline. Total fat and saturated fat also decreased, and protein increased for the replacement of a combination of 2 oz eq/day of protein foods and 3 oz/day of refined grains with 2.0 cups/day of pulses. Additionally, phosphorus increased in the substitution scenarios involving the combinations of 2 oz/day of protein foods and 1.5 and 2.0 oz eq/day of refined grain, and 4 oz eq/day of protein foods and 2.0 and 2.5 oz eq/day of refined grain; zinc increased in 2 oz eq/day of protein foods and 3.0 oz eq/day of the refined grain substitution scenario; vitamin D decreased in all modeling scenarios except for the substitution of 2 oz eq/day of protein foods and 1 oz eq/day of the refined grain combination; and choline decreased for the substitution of 4 oz eq/day of protein foods and 1 to 2 oz eq/day refined grain (Table 4).

The substitution of protein (alone or in combination with refined grains) with pulses were also associated with cost savings whereas the substitution of refined grains with pulses were associated with additional costs (Table 2, Table 3 and Table 4).

## 4. Discussion

The results of the present dietary modeling analysis indicate an additional 1.5 to 2 cups of pulses per week replacing protein foods would increase fiber and decrease cholesterol by more than 10% from the baseline while higher amounts of pulses replacing refined grains or combinations of protein foods and refined grains would consistently increase fiber, iron, magnesium, potassium, and copper in the 2000 kcal Healthy U.S.-Style Pattern. 

Replacing pulses for current protein sources only increased dietary fiber and decreased cholesterol when larger amounts were replaced. There were changes in the energy intake and in other nutrients, but those changes were less than 10% of the bench mark. Dietary fiber is an essential and important component of a healthy diet and is associated with several health benefits [28]. However, more than 90 percent of women and 97 percent of men do not meet the recommended intakes for dietary fiber and DGA has identified it as a dietary component of public health concern for the general U.S. population because low intakes are associated with health concerns [21]. In the present modeling analysis, 1.5–2.0 cups of pulses per week were needed to improve the fiber intake, which equates to about a ¼ cup of pulses per day. We think providing information about how much beans and peas to consume on a daily basis is likely to be better received by consumers, which may help with behavioral changes to consume more pulses (e.g., its only ¼ c per day). Replacing higher amounts of pulses for refined grains or combinations of protein foods and refined grains consistently increased fiber, iron, magnesium, potassium, and copper by more than 10% from the baseline. Potassium and iron (especially for pregnant women) are also identified as a nutrient of public health concern [21]. However similarly, at a higher level of pulses replacing combinations of protein foods and refined grain, while there were also consistent increases in fiber, iron, magnesium, potassium, and copper, there were also decreases in cholesterol, sodium, certain vitamins such as niacin, vitamin D, and vitamin B12, and minerals such as selenium. The current intakes of vitamin D are low and it has been identified as a nutrient of public health concern [21]. The addition of foods high in vitamin D such as mushrooms, which are natural sources of the precursor of vitamin D [29], to the diets could help improve the vitamin D intake. The replacement of refined grains alone or in combination with protein foods with higher amounts of pulses (especially over 1.5 cups/day) resulted in an almost 100% increase in dietary fiber. The USDA reported the fiber intake in the 2000 kcal Healthy U.S.-Style Pattern diet is 30 g/day; however, across the fifteen dietary patterns reported ranging from 1000–3200 kcal/day, the fiber intake was 14–47 g/day [23]. High amounts of dietary fiber could potentially have unpleasant consequences such as abdominal discomforts including bloating, cramping, and flatulence [30,31]. While some consumers may not be able to tolerate the higher fiber values in some of our modeling scenarios, Table 5 shows a summary of some reasonable replacement options of pulses and indicates that an additional 1.0 to 1.5 cups per day of pulses may provide reasonable increases in fiber and other nutrients.

Current DGA recommends including 1.5 cups of beans and peas (pulses) per week as part of the vegetable group for a 2000 kcal healthy diet [21]. DGA also indicated that beans, peas, and lentils may be thought of as either a vegetable or a protein food when aiming to meet the recommended intakes since they have a similar nutrient profile to the foods in both the vegetable group and the protein foods group [21]. However, the 2005 dietary guidelines advisory committee conducted a comprehensive review of the literature and acknowledged that legumes along with dark green vegetables were relatively high in the nutrients needed to meet the unmet nutrient goals, recommending to preferentially increase their amounts and proposed three cups of legumes/week [32]. Subsequently, the Dietary Guidelines 2005 [33] also recommended three cups of legumes/week as part of the vegetable group. However, for unknown reasons, the Dietary Guidelines 2010 and 2015 decreased the recommended amount to 1.5 cups/week as part of healthy dietary patterns [34,35]. While 1.5 cups/week is recommended as part of vegetables in dietary patterns that also include meat (Healthy U.S.-Style Pattern and Healthy Mediterranean Style Pattern), an additional amount of 6 oz eq beans, peas, and lentils per week is recommended as part of the protein foods in the Healthy Vegetarian Style Pattern [21,23]. The NHLBI’s DASH (the Dietary Approaches to Stop Hypertension) diet recommends the consumption of 4 to 5 servings (one serving = 0.5 cup) of nuts, seeds, and legumes per week [36]. Regular consumption of 100 g/day or 0.5 cups/day (50 g of dried beans, lentils, and peas; 25 g of soybeans; and 25 g of peanuts) as part of nutritious and environmentally sustainable dietary patterns was also recommended by the recent Eat Lancet Commission Planetary Health Diet [24]. In multiple systematic reviews, 2 to 3.5 cups per week (1/2 cup or 100 g per day) was found to be the optimal intake amount associated with a positive health outcome [8,17,37,38].

There has been a consistent and ongoing debate in the scientific community to increase the proportion of plant-based foods (including plant protein foods) in the diets to enhance environmental sustainability and to decrease the chronic disease risk [24,39]. Sustainable diets are diets with low environmental impacts, contributing to food security and healthy life, protecting biodiversity and ecosystems, and are affordable and nutritionally adequate [40]. Plant-based diets are reported to be more sustainable and have a lower environmental impact [41]. In a recent prospective cohort study, healthier plant-based dietary patterns containing legumes were associated with a lower environmental impact and a decreased CVD risk [42]. Legumes and pulses are sustainable plant-based foods [19,43] as they contribute to lower greenhouse gas emissions [44], are water efficient [45], and are beneficial for biodiversity and soil health [46]. Our results show that pulses are a more affordable source of dietary protein than the current mix of dietary protein in the Healthy U.S.-Style Pattern.

Future research might include modeling increases in pulses using large national representative databases like the NHNAES in the U.S. to assess the specific impact to various sub-groups of the population (e.g., by race/ethnicity, by age/gender groups, by socioeconomic status, etc.). Continual monitoring of the current intake of pulses also seems meritorious, especially given the recent interest in increasing plant-based foods.

The major strengths of our study include the use of the USDA approach on dietary modeling, leveraging pulses’ contribution to both protein and carbohydrates (e.g., modeling simultaneous changes in protein and refined grains), and converting to possibly more communicable metrics (per day rather than per week). The major limitations of this study were some dietary modeling scenarios call for a large increase in pulses which may not be achievable for some consumers, though vegetarians do tolerate much higher levels of pulses in their diet; and we only highlighted changes of at least 10% or more, though smaller changes might also be useful. Additionally, as in any dietary modeling study, our results evaluated the maximum effect of dietary modeling and may not reflect actual individual dietary behavior.

## 5. Conclusions

In conclusion, the results of this modeling study provide insight into the nutritional benefits of adding pulses as a replacement for the servings of protein foods and/or refined grains in the 2000 kcal Healthy U.S.-Style Pattern. Depending on the amount, the addition of pulses replacing protein foods, refined grains, or combinations of protein foods and refined grains increase fiber, iron, magnesium, potassium, and copper in the 2000 kcal Healthy U.S.-Style Pattern. Our results suggest that encouraging increased pulse consumption may be an effective strategy for improving diet quality and achieving a healthier dietary pattern. However, observational and controlled feeding trials are needed confirm the results of our dietary modeling study.

## Figures and Tables

**Table 1:** Nutrients in 2000 kcal Healthy U.S.-Style Pattern, pulses, protein foods, and refined grains.

	2000 kcal Healthy U.S.-Style Pattern	Pulses (1 Cup)	Protein Foods (1 oz eq)	Refined Grains (1 oz eq)
**Macronutrients**				
Energy (kcal)	2001	242.69	54.29	86.4
Protein (g)	92	15.80	6.69	2.36
Carbohydrate (g)	259	43.62	0.61	16.20
Dietary fiber (g)	30	15.24	0.14	0.71
Total fat (g)	71	1.26	2.72	1.28
Cholesterol (mg)	214	0.00	34.39	0.40
Saturated fatty acids (g)	18	0.24	0.67	0.33
**Minerals**				
Calcium (mg)	1278	77.73	9.73	23.60
Iron (mg)	14	4.21	0.47	0.96
Magnesium (mg)	358	94.33	9.58	7.77
Phosphorus (mg)	1654	267.66	65.32	38.64
Potassium (mg)	3390	723.00	83.07	32.89
Sodium (mg)	1658	0.13	68.78	104.49
Zinc (mg)	13	1.88	0.76	0.23
Copper (mg)	1.4	0.38	0.05	0.04
Selenium (µg)	113	6.46	7.73	6.30
**Vitamins**				
Vitamin A, RAE (µg)	898	0.00	14.37	8.26
Vitamin E, ATE (µg)	10	0.97	0.35	0.12
Vitamin D (IU)	300	0.00	12.48	2.16
Vitamin C (mg)	129	1.11	0.05	0.52
Thiamin (mg)	1.8	0.34	0.04	0.16
Riboflavin (mg)	2.0	0.11	0.07	0.08
Niacin (mg)	23	0.77	1.68	1.22
Vitamin B_6_ (mg)	2.2	0.29	0.10	0.04
Vitamin B_12_ (µg)	6.2	0.00	0.39	0.05
Choline (mg)	355	50.56	33.23	3.39
Vitamin K (µg)	140	5.80	0.38	0.59

Nutrient profile of pulses was computed by using USDA’s beans and peas composite (nutritional profile obtained from Food Pattern Modeling Report [23]), proportionally subtracting the nutritional contribution of edamame (1.3%, nutritional profile obtained from FoodData Central, FCD ID: 2342911) [25] and adjusting calories from rest of the composite. Nutritional profile of USDA’s protein foods composite was computed by proportionally adding nutrients of each individual components (38.21% Red meats, 29.39% Poultry, 2.71% High omega 3 fish, 5.95% Low omega 3 fish, 9.44% Eggs, 1.78% Soy Products, and 12.51% Nuts/Seeds). Nutritional profiles for each component of USDA’s protein foods composite and of Refined grains were obtained from Food Patterns Modeling Report [23]. RAE, retinol activity equivalents; ATE, alpha tocopherol equivalents; IU, international units.

**Table 2:** Effect of replacing 1 to 8 oz eq/week protein foods (PF) with 0.25 to 2.0 cups per week pulses in 2000 kcal Healthy U.S.-Style Pattern (baseline).

	Baseline	After Addition of 0.25 Cups/Week Pulses Replacing 1 oz eq/Week PF	After Addition of 0.5 Cups/Week Pulses Replacing 2 oz eq/Week PF	After Addition of 1.0 Cups/Week Pulses Replacing 4 oz eq/Week PF	After Addition of 1.5 Cups/Week Pulses Replacing 6 oz eq/Week PF	After Addition of 2.0 Cups/Week Pulses Replacing 8 oz eq/Week PF
**Macronutrients**						
Energy (kcal)	2001	2001.91	2002.82	2004.64	2006.47	2008.29
Protein (g)	92	91.61	91.22	90.44	89.65	88.87
Carbohydrate (g)	259	260.47	261.94	264.89	267.83	270.77
Dietary fiber (g)	30	30.52	31.05	32.09	33.14 *	34.19 *
Total fat (g)	71	70.66	70.31	69.63	68.94	68.25
Cholesterol (mg)	214	209.09	204.18	194.35	184.53 *	174.70 *
Saturated fatty acids (g)	18	17.91	17.83	17.65	17.48	17.31
**Minerals**						
Calcium (mg)	1278	1279.39	1280.77	1283.54	1286.32	1289.09
Iron (mg)	14	14.08	14.17	14.33	14.50	14.67
Magnesium (mg)	358	360.00	362.00	366.00	370.00	374.00
Phosphorus (mg)	1654	1654.23	1654.46	1654.91	1655.37	1655.82
Potassium (mg)	3390	3403.95	3417.91	3445.82	3473.73	3501.63
Sodium (mg)	1658	1648.18	1638.36	1618.72	1599.08	1579.44
Zinc (mg)	13	12.96	12.92	12.84	12.76	12.67
Copper (mg)	1.4	1.41	1.41	1.43	1.44	1.45
Selenium (µg)	113	112.13	111.25	109.51	107.76	106.01
**Vitamins**						
Vitamin A, RAE (µg)	898	895.95	893.90	889.79	885.69	881.58
Vitamin E, ATE (µg)	10	9.98	9.97	9.94	9.91	9.88
Vitamin D (IU)	300	298.22	296.44	292.87	289.31	285.74
Vitamin C (mg)	129	129.03	129.06	129.13	129.19	129.26
Thiamin (mg)	1.8	1.81	1.81	1.83	1.84	1.85
Riboflavin (mg)	2	1.99	1.99	1.97	1.96	1.95
Niacin (mg)	23	22.79	22.58	22.15	21.73	21.30
Vitamin B_6_ (mg)	2.2	2.20	2.19	2.18	2.18	2.17
Vitamin B_12_ (µg)	6.2	6.14	6.09	5.97	5.86	5.75
Choline (mg)	355	352.06	349.12	343.23	337.35	331.47
Vitamin K (µg)	140	140.15	140.31	140.61	140.92	141.22
**Dietary cost**						
Cost, differential ($)		−0.04	−0.08	−0.15	−0.23	−0.30

Baseline nutritional profiles of 2000 kcal Heathy U.S.-Style Pattern was obtained from Food Pattern Modeling Report [23]. Nutrient profiles after replacing protein foods (PF) with pulses were computed by removing the nutrients of protein foods and adding the nutrients of beans and peas (Table 1) from the nutrients of the 2000 kcal Healthy U.S.-Style Pattern. Since 2000 kcal Healthy U.S.-Style Pattern includes 1.5 cups/week of pulses this additional 0.25 to 2.0 cups/week of would increase total amount of pulses to 1.75 to 3.5 cups/week. * Indicates ≥10% change from baseline; values in bold indicate increase and values in italics indicate decrease from baseline. RAE, retinol activity equivalents; ATE, alpha tocopherol equivalents; IU, international units.

**Table 3:** Effect of replacing 1 to 4 oz eq/day refined grains (RG) with 0.5 to 2.0 cups/day pulses in 2000 kcal Healthy U.S.-Style Pattern (baseline).

	Baseline	After Addition of 0.5 Cups/Day Pulses Replacing 1 oz eq/Day RG	After Addition of 1.0 Cups/Day Pulses Replacing 2 oz eq/Day RG	After Addition of 1.5 Cups/Day Pulses Replacing 3 oz eq/Day RG	After Addition of 2.0 Cups/Day Pulses Replacing 4 oz eq/Day RG
**Macronutrients**					
Energy (kcal)	2001	2035.95	2070.89	2105.84	2140.78
Protein (g)	92	97.54	103.08 *	108.62 *	114.16 *
Carbohydrate (g)	259	264.61	270.22	275.84	281.45
Dietary fiber (g)	30	36.91 *	43.82 *	50.73 *	57.64 *
Total fat (g)	71	70.35	69.70	69.05	68.40
Cholesterol (mg)	214	213.60	213.20	212.80	212.40
Saturated fatty acids (g)	18	17.79	17.58	17.36	17.15
**Minerals**					
Calcium (mg)	1278	1293.27	1308.53	1323.80	1339.07
Iron (mg)	14	15.15	16.29 *	17.44 *	18.59 *
Magnesium (mg)	358	397.40 *	436.79 *	476.19 *	515.58 *
Phosphorus (mg)	1654	1749.19	1844.38 *	1939.56 *	2034.75 *
Potassium (mg)	3390	3718.61 *	4047.22 *	4375.83 *	4704.44 *
Sodium (mg)	1658	1553.58	1449.15 *	1344.73 *	1240.31 *
Zinc (mg)	13	13.71	14.42 *	15.14 *	15.85 *
Copper (mg)	1.4	1.55 *	1.70 *	1.85 *	2.00 *
Selenium (µg)	113	109.93	106.86	103.79	100.72 *
**Vitamins**					
Vitamin A, RAE (µg)	898	889.74	881.48	873.22	864.96
Vitamin E, ATE (µg)	10	10.37	10.73	11.10 *	11.46 *
Vitamin D (IU)	300	297.84	295.68	293.52	291.36
Vitamin C (mg)	129	129.04	129.07	129.11	129.15
Thiamin (mg)	1.8	1.81	1.82	1.83	1.84
Riboflavin (mg)	2	1.97	1.95	1.92	1.90
Niacin (mg)	23	22.17	21.33	20.50 *	19.66 *
Vitamin B_6_ (mg)	2.2	2.31	2.41 *	2.52 *	2.62 *
Vitamin B_12_ (µg)	6.2	6.15	6.10	6.05	6.00
Choline (mg)	355	376.89	398.78 *	420.67 *	**442.56**
Vitamin K (µg)	140	142.31	144.62	146.94	149.25
**Dietary cost**					
Cost, differential ($)		0.17	0.34	0.51	0.67

Baseline nutritional profiles of 2000 kcal Heathy U.S.-Style Pattern was obtained from Food Pattern Modeling Report [23]. Nutrient profiles after replacing protein foods (PF) with pulses were computed by removing the nutrients of protein foods and adding the nutrients of pulses (Table 1) from the nutrients of the 2000 kcal Healthy U.S.-Style Pattern.* Indicates ≥10% change from baseline; values in bold indicate increase and values in italics indicate decrease from baseline. RAE, retinol activity equivalents; ATE, alpha tocopherol equivalents; IU, international units.

**Table 4:** Effect of replacing combinations of protein foods (PF) and refined grains (RG) with pulses in 2000 kcal Healthy U.S.-Style Pattern.

	Baseline	After Addition of 1.0 Cups/Day Pulses Replacing 2 oz eq/day PF & 1 oz eq/Day RG	After Addition of 1.5 Cups/Day Pulses Replacing 2 oz eq/day PF & 2 oz eq/Day RG	After Addition of 2.0 Cups/Day Pulses Replacing 2 oz eq/day PF & 3 oz eq/Day RG	After Addition of 1.5 Cups/Day Pulses Replacing 4 oz eq/Day PF & 1 oz eq/Day RG	After Addition of 2.0 Cups/Day Pulses Replacing 4 oz eq/Day PF & 2 oz eq/Day RG	After Addition of 2.5 Cups/Day Pulses Replacing 4 oz eq/Day PF & 3 oz eq/Day RG
**Macronutrients**							
Energy (kcal)	2001	2048.70	2083.65	2118.59	2061.46	2096.40	2131.35
Protein (g)	92	92.07	97.61	103.15 *	86.60	92.14	97.68
Carbohydrate (g)	259	285.21 *	290.83 *	296.44 *	305.81 *	311.43 *	317.04 *
Dietary fiber (g)	30	44.24 *	51.15 *	58.06 *	51.57 *	58.48 *	65.39 *
Total fat (g)	71	65.54	64.89	64.24 *	60.73 *	60.08 *	59.43 *
Cholesterol (mg)	214	144.83 *	144.43 *	144.03 *	76.06 *	75.66 *	75.26 *
Saturated fatty acids (g)	18	16.58	16.36	16.15 *	15.36 *	15.15 *	14.94 *
**Minerals**							
Calcium (mg)	1278	1312.67	1327.94	1343.21	1332.08	1347.35	1362.62
Iron (mg)	14	16.32 *	17.46 *	18.61 *	17.49 *	18.63 *	19.78 *
Magnesium (mg)	358	425.40 *	464.79 *	504.19 *	453.40 *	492.80 *	532.19 *
Phosphorus (mg)	1654	1752.38	1847.57 *	1942.76 *	1755.57	1850.76 *	1945.95 *
Potassium (mg)	3390	3913.97 *	4242.58 *	4571.19 *	4109.33 *	4437.94 *	4766.55 *
Sodium (mg)	1658	1416.09 *	1311.67 *	1207.24 *	1278.60 *	1174.18 *	1069.76 *
Zinc (mg)	13	13.14	13.86	14.57 *	12.57	13.29	14.00
Copper (mg)	1.4	1.64 *	1.79 *	1.93 *	1.73 *	1.87 *	2.02 *
Selenium (µg)	113	97.70 *	94.63 *	91.56 *	85.47 *	82.40 *	79.33 *
**Vitamins**							
Vitamin A, RAE (µg)	898	861.01	852.75	844.49	832.28	824.02	815.76
Vitamin E, ATE (µg)	10	10.15	10.52	10.89	9.94	10.31	10.67
Vitamin D (IU)	300	272.89	270.73 *	268.57 *	247.93 *	245.77 *	243.61 *
Vitamin C (mg)	129	129.49	129.52	129.56	129.94	129.98	130.01
Thiamin (mg)	1.8	1.90	1.91	1.92	2.00 *	2.01 *	2.02 *
Riboflavin (mg)	2	1.89	1.86	1.83	1.80 *	1.77 *	1.75 *
Niacin (mg)	23	19.19 *	18.36 *	17.53 *	16.22 *	15.39 *	14.55 *
Vitamin B_6_ (mg)	2.2	2.25	2.35	2.46 *	2.19	2.30	2.40
Vitamin B_12_ (µg)	6.2	5.36 *	5.31 *	5.26 *	4.57 *	4.52 *	4.47 *
Choline (mg)	355	335.71	357.60	379.49	294.52 *	316.41 *	338.30
Vitamin K (µg)	140	144.45	146.76	149.07	146.59	148.90	151.21
**Dietary Cost**							
Cost, differential ($)		−0.36	−0.20	−0.03	−0.90	−0.73	−0.56

Baseline nutritional profiles of 2000 kcal Heathy U.S.-Style Pattern was obtained from Food Pattern Modeling Report [23]. Nutrient profiles after replacing combinations of protein foods (PF) and refined grains (RG) with pulses were computed by removing the nutrients of protein foods and refined grains and adding the nutrients of pulses (Table 1) from the nutrients of the 2000 kcal Healthy U.S.-Style Pattern.* Indicates ≥10% change from baseline; values in bold indicate increase and values in italics indicate decrease from baseline. RAE, retinol activity equivalents; ATE, alpha tocopherol equivalents; IU, international units.

**Table 5:** Effect of some options of replacing protein foods (PF) and/or refined grains (RG) with pulses in 2000 kcal Healthy U.S.-Style Pattern.

	% Change after Addition of 1.5 Cups per Week Pulses Replacing 6 oz eq/Week PF	% Change after Addition of 1.0 Cups per Day pulses Replacing 2 oz eq/Day RG	% Change after Addition of 1.0 Cup per Day Pulses Replacing 2 oz eq/Day PF & 1 oz eq/Day RG
**Macronutrients**			
Energy	0	3	2
Protein	−3	12	0
Carbohydrate	3	4	10
Dietary fiber	10	46	47
Total Fat	−3	−2	−8
Cholesterol	−14	0	−32
Saturated fatty acids	−3	−2	−8
**Minerals**			
Calcium	1	2	3
Iron	4	16	17
Magnesium	3	22	19
Phosphorus	0	12	6
Potassium	2	19	15
Sodium	−4	−13	−15
Zinc	−2	11	1
Copper	3	21	17
Selenium	−5	−5	−14
**Vitamins**			
Vitamin A, RAE	−1	−2	−4
Vitamin E, ATE	−1	7	2
Vitamin D	−4	−1	−9
Vitamin C	0	0	0
Thiamin	2	1	6
Riboflavin	−2	−3	−6
Niacin	−6	−7	−17
Vitamin B_6_	−1	10	2
Vitamin B_12_	−5	−2	−14
Choline	−5	12	−5
Vitamin K	1	3	3

% changes are calculated from data in Table 2, Table 3 and Table 4.

## Data Availability

All data obtained for this study are publicly available at: https://www.dietaryguidelines.gov/sites/default/files/2020-07/FoodPatternModeling_Report_2YearsandOlder.pdf; https://fdc.nal.usda.gov/ (accessed on 4 April 2023).

## Supplementary Materials

The following supporting information can be downloaded at: https://www.mdpi.com/article/10.3390/nu15204355/s1, Table S1: Cups per day of pulses in different modeling scenarios.

## References

[1] Food and Agricultural Organization (2023). World Pulse Day. https://www.fao.org/world-pulses-day/en/.

[2] Food and Agricultural Organization Global Forum on Food Security and Nutrition: Pulses are Praised for Their Health, Environmental and Economic Benefits. How Can Their Full Potential be Tapped? Discussion No. 128—30.05.2016–19.06.2016. https://www.fao.org/3/bl626e/bl626e.pdf.

[3] Marinangeli C.P.F., Curran J., Barr S.I., Slavin J., Puri S., Swaminathan S., Tapsell L., Patterson C.A. (2017). Enhancing nutrition with pulses: Defining a recommended serving size for adults. Nutr. Rev..

[4] Didinger C., Thompson H.J. (2021). Defining Nutritional and Functional Niches of Legumes: A Call for Clarity to Distinguish a Future Role for Pulses in the Dietary Guidelines for Americans. Nutrients.

[5] Mudryj A.N., Yu N., Aukema H.M. (2014). Nutritional and health benefits of pulses. Appl. Physiol. Nutr. Metab..

[6] Bouchenak M., Lamri-Senhadji M. (2013). Nutritional quality of legumes, and their role in cardiometabolic risk prevention: A review. J. Med. Food.

[7] Mitchell D.C., Marinangeli C.P.F., Pigat S., Bompola F., Campbell J., Pan Y., Curran J.M., Cai D.J., Jaconis S.Y., Rumney J. (2021). Pulse Intake Improves Nutrient Density among US Adult Consumers. Nutrients.

[8] Ha V., Sievenpiper J.L., de Souza R.J. (2014). Effect of dietary pulse intake on established therapeutic lipid targets for cardiovascular risk reduction: A systematic review and meta-analysis of randomized controlled trials. CMAJ.

[9] Marventano S., Izquierdo Pulido M., Sánchez-González C., Godos J., Speciani A., Galvano F., Grosso G. (2017). Legume consumption and CVD risk: A systematic review and meta-analysis. Pub. Health Nutr..

[10] Jayalath V.H., de Souza R.J., Sievenpiper J.L. (2014). Effect of dietary pulses on blood pressure: A systematic review and meta-analysis of controlled feeding trials. Am. J. Hypertens..

[11] Viguiliouk E., Blanco Mejia S., Kendall C.W., Sievenpiper J.L. (2017). Can pulses play a role in improving cardiometabolic health? Evidence from systematic reviews and meta-analyses. Ann. N. Y. Acad. Sci..

[12] Zhu B., Sun Y., Qi L., Zhong R., Miao X. (2015). Dietary legume consumption reduces risk of colorectal cancer: Evidence from a meta-analysis of cohort studies. Sci. Rep..

[13] Li J., Mao Q.Q. (2017). Legume intake and risk of prostate cancer: A meta-analysis of prospective cohort studies. Oncotarget.

[14] Zhong X.S., Ge J., Chen S.W., Xiong Y.Q., Ma S.J., Chen Q. (2018). Association between Dietary Isoflavones in Soy and Legumes and Endometrial Cancer: A Systematic Review and Meta-Analysis. J. Acad. Nutr. Diet..

[15] Sievenpiper J.L., Kendall C.W., Esfahani A. (2009). Effect of non-oil-seed pulses on glycaemic control: A systematic review and meta-analysis of randomised controlled experimental trials in people with and without diabetes. Diabetologia.

[16] Li S.S., Kendall C.W., de Souza R.J., Jayalath V.H., Cozma A.I., Ha V., Mirrahimi A., Chiavaroli L., Augustin L.S., Blanco Mejia S. (2014). Dietary pulses, satiety and food intake: A systematic review and meta-analysis of acute feeding trials. Obesity.

[17] Kim S.J., de Souza R.J., Choo V.L., Ha V., Cozma A.I., Chiavaroli L., Mirrahimi A., Blanco Mejia S., Di Buono M., Bernstein A.M. (2016). Effects of dietary pulse consumption on body weight: A systematic review and meta-analysis of randomized controlled trials. Am. J. Clin. Nutr..

[18] Zargarzadeh N., Mousavi S.M., Santos H.O., Aune D., Hasani-Ranjbar S., Larijani B., Esmaillzadeh A. (2023). Legume Consumption and Risk of All-Cause and Cause-Specific Mortality: A Systematic Review and Dose-Response Meta-Analysis of Prospective Studies. Adv. Nutr..

[19] McDermott J., Wyatt A.J. (2017). The role of pulses in sustainable and healthy food systems. Ann. N. Y. Acad. Sci..

[20] Hughes J., Pearson E., Grafenauer S. (2022). Legumes—A Comprehensive Exploration of Global Food-Based Dietary Guidelines and Consumption. Nutrients.

[21] U.S. Department of Agriculture, U.S. Department of Health and Human Services (2020). Dietary Guidelines for Americans, 2020–2025.

[22] U.S. Department of Agriculture Choose My Plate. https://www.choosemyplate.gov.

[23] 2020 Dietary Guidelines Advisory Committee and Food Pattern Modeling Team (2020). Food Pattern Modeling: Ages 2 Years and Older. 2020 Dietary Guidelines Advisory Committee Project.

[24] Willett W., Rockström J., Loken B., Springmann M., Lang T., Vermeulen S., Garnett T., Tilman D., DeClerck F., Wood A. (2019). Food in the Anthropocene: The EAT-Lancet Commission on healthy diets from sustainable food systems. Lancet.

[25] USDA, ARS (2019). FoodData Central. https://fdc.nal.usda.gov/.

[26] U.S. Department of Agriculture (2023). Economic Research Service. Purchase to Plate Suite. https://www.ers.usda.gov/data-products/purchase-to-plate/.

[27] U.S. Department of Agriculture, Agriculture Research Service USDA Food Patterns Equivalents Database. https://www.ars.usda.gov/northeast-area/beltsville-md-bhnrc/beltsville-human-nutrition-research-center/food-surveys-research-group/docs/fped-overview/.

[28] Anderson J.W., Baird P., Davis R.H.J., Ferreri S., Knudtson M., Koraym A., Waters V., Williams C.L. (2009). Health benefits of dietary fiber. Nutr. Rev..

[29] Agarwal S., Fulgoni V.L. (2021). Nutritional impact of adding a serving of mushrooms to USDA Food Patterns—A dietary modeling analysis. Food Nutr. Res..

[30] Borkoles E., Krastins D., van der Pols J.C., Sims P., Polman R. (2022). Short-Term Effect of Additional Daily Dietary Fibre Intake on Appetite, Satiety, Gastrointestinal Comfort, Acceptability, and Feasibility. Nutrients.

[31] de Vries J., Miller P.E., Verbeke K. (2015). Effects of cereal fiber on bowel function: A systematic review of intervention trials. World J. Gastroenterol..

[32] US Department of Health and Human Services (2005). The Report of the Dietary Guidelines Advisory Committee on Dietary Guidelines for Americans. https://www.dietaryguidelines.gov/sites/default/files/2019-10/FINAL2005DGACReport.pdf.

[33] United States Department of Agriculture and U.S., Department of Health and Human Services Dietary Guidelines for Americans 2005.

[34] United States Department of Agriculture and U.S., Department of Health and Human Services Dietary Guidelines for Americans 2010.

[35] United States Department of Agriculture and U.S., Department of Health and Human Services Dietary Guidelines for Americans 2015–2020.

[36] NIH, NHLBI (2021). DASH Eating Plan. https://www.nhlbi.nih.gov/education/dash-eating-plan.

[37] Viguiliouk E., Glenn A.J., Nishi S.K., Chiavaroli L., Seider M., Khan T., Bonaccio M., Iacoviello L., Mejia S.B., Jenkins D.J.A. (2019). Associations between Dietary Pulses Alone or with Other Legumes and Cardiometabolic Disease Outcomes: An Umbrella Review and Updated Systematic Review and Meta-analysis of Prospective Cohort Studies. Adv. Nutr..

[38] Afshin A., Micha R., Khatibzadeh S., Mozaffarian D. (2014). Consumption of nuts and legumes and risk of incident ischemic heart disease, stroke, and diabetes: A systematic review and meta-analysis. Am. J. Clin. Nutr..

[39] IOM (2014). Sustainable Diets: Food for Healthy People and a Healthy Planet.

[40] FAO (2012). Sustainable Diets and Biodiversity: Directions and Solutions for Policy, Research and Action. http://www.fao.org/3/a-i3004e.pdf2012.

[41] Carey C.N., Paquette M., Sahye-Pudaruth S., Dadvar A., Dinh D., Khodabandehlou K., Liang F., Mishra E., Sidhu M., Brown R. (2023). The Environmental Sustainability of Plant-Based Dietary Patterns: A Scoping Review. J. Nutr..

[42] Musicus A.A., Wang D.D., Janiszewski M., Eshel G., Blondin S.A., Willett W., Stampfer M.J. (2022). Health and environmental impacts of plant-rich dietary patterns: A US prospective cohort study. Lancet Planet Health.

[43] Cusworth G., Garnett T., Lorimer J. (2021). Legume dreams: The contested futures of sustainable plant-based food systems in Europe. Glob. Environ. Chang..

[44] Springmann M., Clark M., Mason-D’Croz D., Wiebe K., Bodirsky B.L., Lassaletta L., de Vries W., Vermeulen S.J., Herrero M., Carlson K.M. (2018). Options for keeping the food system within environmental limits. Nature.

[45] Mekonnen M.M., Hoekstra A.Y. (2012). A global assessment of the water footprint of farm animal products. Ecosystems.

[46] Gan Y., Liang C., Hamel C., Cutforth H., Wang H., Zentner R., Campbell C.A. (2015). Soil and crop responses to different pulse crops and their impact on subsequent cereal crops in rotation. Agric. Ecosyst. Environ..

