# Supplementary material for: Effect of Adding Pulses to Replace Protein Foods and Refined Grains in Healthy Dietary Patterns
Source: Nutrients. 2023 Oct 13;15(20):4355. doi: 10.3390/nu15204355 (PMC10610119; doi:10.3390/nu15204355)
Supplement: Supplementary file 1 [file nutrients-15-04355-s001.zip › nutrients-2628803-supplementary.pdf]

Supplemental Table S1. Cups per day of pulses in different modeling scenarios.

Modeling Scenario number	Modeling Scenario details	Pulses (cups/day)
1A	Addition of 0.25 cups/week Pulses replacing 1 oz eq/week Protein Foods	0.04
1B	Addition of 0.5 cups/week Pulses replacing 2 oz eq/week Protein Foods	0.07
1C	Addition of 1.0 cups/week Pulses replacing 4 oz eq/week Protein Foods	0.14
1D	Addition of 1.5 cups/week Pulses replacing 6 oz eq/week Protein Foods	0.21
1E	Addition of 2.0 cups/week Pulses replacing 8 oz eq/week Protein Foods	0.29
2A	Addition of 0.5 cups/day Pulses replacing 1 oz eq/day Refined Grains	0.50
2B	Addition of 1.0 cups/day Pulses replacing 2 oz eq/day Refined Grains	1.00
2C	Addition of 1.5 cups/day Pulses replacing 3 oz eq/day Refined Grains	1.50
2D	Addition of 2.0 cups/day Pulses replacing 4 oz eq/day Refined Grains	2.00
3A	Addition of 1.0 cups/day Pulses replacing 2 oz/day Protein Foods and 1 oz/d Refined Grains	1.00
3B	Addition of 1.5 cups/day Pulses replacing 2 oz/day Protein Foods and 2 oz/d Refined Grains	1.50
3C	Addition of 2.0 cups/day Pulses replacing 2 oz/day Protein Foods and 3 oz/d Refined Grains	2.00
4A	Addition of 1.5 cups/day Pulses replacing 4 oz/day Protein Foods and 1 oz/d Refined Grains	1.50
4B	Addition of 2.0 cups/day Pulses replacing 4 oz/day Protein Foods and 2 oz/d Refined Grains	2.00
4C	After addition of 2.5 cups/day Pulses replacing 4 oz/day Protein Foods and 3 oz/d Refined Grains	2.50
